# Psychological health correlation of express delivery workers’ occupational stress in the information logistics environment

**DOI:** 10.3389/fpsyg.2022.975387

**Published:** 2022-08-11

**Authors:** Meishun Lin

**Affiliations:** School of E-Commerce and Logistics, Suzhou Institute of Trade and Commerce, Suzhou, China

**Keywords:** mental health, occupational stress, employee health, express delivery industry, information logistics environment

## Abstract

With the promotion of the Internet of Things technology, more and more industries have begun to combine with the Internet of Things technology. After joining the WTO, China’s market economy has continued to deepen. During this period, the e-commerce industry has developed rapidly, which has promoted the rise of the express delivery industry. While the rise of the industry provides jobs for employees, it also brings enormous pressure to employees. Due to the occupational stress of various stressors in the express delivery industry, a series of psychological problems have been caused. “Occupational stress” is also called occupational stress, which refers to the physiological and psychological stress caused by the imbalance between objective needs and personal adaptability in a certain occupational environment. The purpose of this paper is to conduct a correlation study on the psychological health of express delivery workers’ occupational stress in the information logistics environment. It is expected to explore the relationship between occupational stress and mental health of couriers in the context of informatization, so as to provide employees with a better working environment and reduce personal stress. Logistics informatization means that logistics enterprises use modern information technology to collect, classify, transmit, summarize, identify, track, and query all or part of the information generated in the logistics process, so as to realize the control of the flow of goods. This paper analyzed the inventory of express companies, and reasonably analyzed the profit composition of the inventory link, then used a more reasonable time series seasonal coefficient method to predict the amount of incoming shipments. This paper used a combination of theoretical and empirical methods to discuss the psychological health and occupational stress of express delivery workers and to explore the relationship between the two. The experiment results of this paper showed that in the interpersonal sensitivity analysis, the coefficient of occupational stress is 0.052, and the degree of occupational stress is proportional to interpersonal sensitivity. The higher the occupational stress, the higher the interpersonal sensitivity index. The coefficient of occupational stress is-0.31. Occupational stress is inversely proportional to age. The older the age, the lower the interpersonal sensitivity index.

## Introduction

The express delivery industry emerged in China in the 1970s. With the advancement of technology, the business model of the express delivery industry is constantly changing. Especially with the promotion of Internet of Things technology, the e-commerce industry has sprung up. E-commerce is a business activity centered on the exchange of commodities with information network technology as the means, and business is the core purpose. In the information logistics environment, express delivery has also become an important part of the industry. While providing a large number of jobs for the society, it also brings great challenges to the staff. Employees need to face high-load work during the holiday promotion time, and add a lot of technology to work operations, which brings challenges to low-educated employees. A large number of work problems have created occupational stress and caused employees to have various psychological problems. Therefore, it is very necessary to explore the occupational stress and mental health of employees in the information logistics environment.

With the continuous development of the economy, different degrees of occupational pressure will appear in all walks of life. Eyimaya aimed to determine the level of stress experienced by railway workers. He surveyed workers in related industries and collected data using a personal information form and a Doetinchem organizational stress questionnaire. The experimental results showed that employees were affected by all sub-dimensions of stressors, social changes, psychological stress, and health complaints, and their stress was at a moderate level. The study found that workers are primarily affected by uncertainty about their responsibilities and future careers. Descriptive characteristics of workers as well as job-related occupational characteristics showed statistically significant differences in mean scores on the subscales of stressors, social variables, psychological variables, and health complaints (Eyimaya and Tezel, 2021). Ahmad aimed to understand the impact of occupational stress, which is divided into four structures, namely job stress, job support, job satisfaction, and job nature. In order to conduct the study, he sent questionnaires to six universities: two in the public sector and four in the private sector. These colleges were selected based on convenience, and respondents to these colleges were selected based on purposeful sampling techniques. He employed correlation and multiple regression as statistical tools to analyze the data. Multiple regression is to study the regression of a dependent variable and two or more independent variables, and it is to reflect the law that the quantity of a phenomenon or thing changes accordingly according to the change of the quantity of multiple phenomena or things. Experimental analysis showed that all variables of occupational stress have a significant impact on employee personality, and the overall model is significant within the 99.9% confidence interval (Ahmad and Ashraf, 2017). Substantial evidence showed the importance of job demands and job resources in predicting employee health and motivational outcomes. However, there is little empirical evidence explaining how broader organizational factors influence these results. Using the job demand-resource (JD-R) model as a theoretical explanation for occupational stress, Raper et al. (2020) examined the impact of employee alignment with their organization’s strategic goals (strategic alignment) in predicting long-term occupational health outcomes. The sample completed two self-report surveys over a 12-month period. The findings suggested that strategic adjustment is the only resource that reduces psychological stress and increases work engagement over time. With time going by, job demands were not found to be significantly associated with psychological stress or work engagement. There is also no evidence that work resources moderate health and motivational outcomes (Raper et al., 2020). The express delivery industry is one of the most important components of modern logistics. At the same time, the express network is one of the most important infrastructures for express companies to participate in the operation. The express network is a special network attached to the transportation network. It is a delivery system that is connected by a number of express call centers, express pick-up and delivery points, transfer stations (distribution centers) at all levels, and transportation routes according to certain principles and methods. Lin proposed a combinatorial optimization model for express delivery network design. The purpose of optimization is to consider the frequency delay accumulated in the sorting process and determine the transportation organization mode of each express flow. He formulated a nonlinear zero–one integer programming model to describe the problem. The objective function of the model is to minimize the total cost including cumulative cost, transportation cost, and transfer cost. Furthermore, he expressed the uniqueness condition of each express flow using a transitive chain implemented by a recursive formula. In addition, the model took into account the limited capacity of the facility. He illustrated the efficiency of the algorithm by taking a real express delivery network in China as an example, and developed a simulated annealing algorithm for obtaining an approximate optimal solution after preprocessing. It obtained acceptable results (Lin et al., 2020). Although these theories address occupational stress, they are not integrated with mental health.

With the rise of psychology, mental health has received increasing attention. Addabbo aimed to study how the degree of wage polarization changes during times of economic crisis, taking into account factors affecting labor heterogeneity. The existence of monopoly industries, flaws in fiscal policy, and imperfect development strategies are all important factors that cause wage differentiation. He considers gender differences in the evolution of social disruption by analyzing men and women separately. The data showed that polarization arises from two trends that lead to the creation of social tensions: homogeneity or cohesion within groups and heterogeneity between groups. From a contract-type perspective, women are more polarized than men. In terms of education level, job status, and occupational status, women’s wages are also more polarized than men’s wages (Addabbo et al., 2018). The profound organizational changes the banking industry has undergone, including changes in working conditions, have led to heightened emotional tensions experienced by its employees. However, the stress levels experienced by the staff of commercial banks and cooperative banks are not the same because the management methods implemented by the two types of banks are different. The purpose of Kamierczyk was to compare the stress levels of employees. He surveyed bank employees who participated in the experiment and used as a background for the analysis. The study found that commercial bank employees had higher stress levels than cooperative bank employees; furthermore, this difference was particularly pronounced among certain employee groups. The enormous pressure to achieve optimal sales performance leads to greater stress among commercial bank employees, and the increased use of information technology and internal competition in the workplace may affect stress levels (Kamierczyk and Zajdler, 2020). The growing interest among scientists in the problem of occupational stress suggests that the phenomenon is becoming more common across the globe. Extensive research has shown that workers in the social services sector are most likely to develop the syndrome due to stressors that can lead to burnout. Rowicka conducted qualitative research to understand pedagogy students’ perceptions of stress-inducing job factors in the teaching profession and ways to cope with organizational stress. The findings showed that respondents identified work-related stressors as a variety of factors related to the work environment, educational reform, and socio-political situation. These factors include the low social status and underpayment of teachers, and the changes brought about by educational reforms that have brought additional tasks and obligations. It led to ambiguity in teachers’ professional roles and job insecurity. Further stress triggers include behavioral problems and challenges in students, lack of motivation to learn, and inappropriate practices of parent, especially their harsh attitudes, aversions, and lack of cooperation between parents and the school. Other stressors include work overload and bureaucracy, adverse working conditions, and barriers to career growth and development. In terms of stress coping strategies, future teachers will propose remedies, such as searching for information and making direct efforts to resolve the problem satisfactorily. Additionally, they choose strategies aimed at regulating emotions and tension. The findings instill optimism as pedagogy students are able to use adaptive coping strategies in the areas of teacher-student relationships and mutual interpersonal communication, which is especially desirable in the teaching profession. More importantly, the ability to relax and relieve stress helps a person to think rationally and provide higher quality work, which is especially important in this socially valuable profession (Rowicka, 2020). Marshall believed that managing women’s volunteering is a new and promising research focus. It could provide insight into how gender works in the voluntary sector when women have authority in the formal labor market. The gender-segregated structure of paid work and the voluntary sector suggested that gender may be an important factor governing women’s decisions about whether, where, and how to volunteer. Existing research showed that gender and authority together make female managers face public and agency challenges. Female managers may engage in volunteering as a form of remedial work to resolve the tension between occupational status and gender. Further research on the motivations and opportunities for managing women’s volunteering, their positions in voluntary organizations, and the impact of volunteering on their occupational status in the formal labor market can contribute to understanding gender and power and enrich academic research on volunteering (Marshall and Taniguchi, 2021). Although these theories address psychological problems, they are less integrated with occupational stress and less practical.

While economic development provides people with jobs, it also brings occupational stress to people. Long-term occupational stress has brought various psychological problems to employees. According to the research, under normal circumstances, the interpersonal sensitivity index of employees is 1.7, and the index of express employees is 2.4; under normal circumstances, the depression index of employees is 1.6, and the index of express employees is 2.2. It can be seen that the express industry is more prone to psychological problems. When exploring the related factors of occupational stress, the coefficient of occupational stress is-0.31, indicating that occupational stress is inversely proportional to age. The older the age, the lower the interpersonal sensitivity index. The working age index is 0.12, indicating that the degree of working age is proportional to the interpersonal sensitivity. To a certain extent, the greater the working age, the higher the interpersonal sensitivity index.

## Methods on mental health of express workers’ occupational stress

Driven by the Internet of Things technology, the number and scale of online shopping are in the process of increasing. In order to ensure the normal operation of express delivery, express delivery companies have divided them into different markets. The industry has shown great market vitality while constantly planning (Kharitonov, 2017; Rasmus et al., 2017). The development of information technology has greatly improved the efficiency of information transmission. With the application of intelligent communication in logistics operations, people are more and more aware of the importance of information to the flow of tangible items. Information resources are not only showing more and more prominent importance for the effective delivery and optimal allocation of material resources, but also providing customers with logistics information such as product availability, delivery schedule, and transportation status has become an integral part of customer service. Especially in the 21st century, the trend of globalization has become more and more obvious, and the scope of freight transportation has become wider and wider. They both force enterprises to establish a complete logistics system to improve work efficiency while ensuring work quality (Shi et al., 2017; Chisuwa et al., 2019).

**Figure 1:**
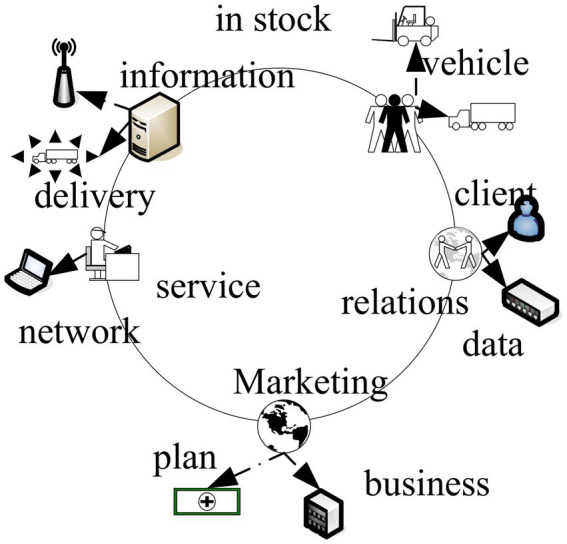
Construction mode of information logistics system.

With the global development of IoT technology, it has brought great changes to traditional logistics (Al-Wahaibi, 2019). In the view of the operation mode of e-commerce, it is necessary to deal with issues such as payment and delivery of goods in the traditional business mode while in the e-commerce mode. Internet technology can be used to provide full service (Berry and Sessions, 2018; Schouten et al., 2019). The core technology of modern logistics is information technology, and information management plays an important role in modern logistics management, maximizing the integration of various steps of logistics and transportation, and facing the logistics needs of society as a whole (Feibert and Jacobsen, 2019). Information technology is a major source of increased productivity and competitiveness. It continuously increases speed and capability while reducing costs. Figure 1 shows the construction mode of the information logistics system.

Common problems in daily life during stress have different effects on the human body according to the specific situation of stress. Tension is an actual perceived imbalance between demands and an individual’s ability to respond, and it is one of the many observable dysfunctions that can be caused when the demands are not met. With the deepening of the detailed research on “nervousness,” it is extended continuously to the workplace (Sol and Rocca, 2017; Lyster, 2019). In fact, there are different views on the understanding of occupational stress at different stages. The common saying is that the normal response of an individual changes abnormally in a specific environment. All in all, occupational stress is the interaction of working conditions and individuals. Working conditions include role factors, job nature, interpersonal relationships, organizational structure of the job, personnel management, and physical factors. According to a series of studies on stress, scholars have proposed a matching model of the subjective and objective environments of occupational stress. If the subjective and objective environments are unbalanced, stress will appear. It can be seen that occupational stress is a dynamic pattern.

**Figure 2:**
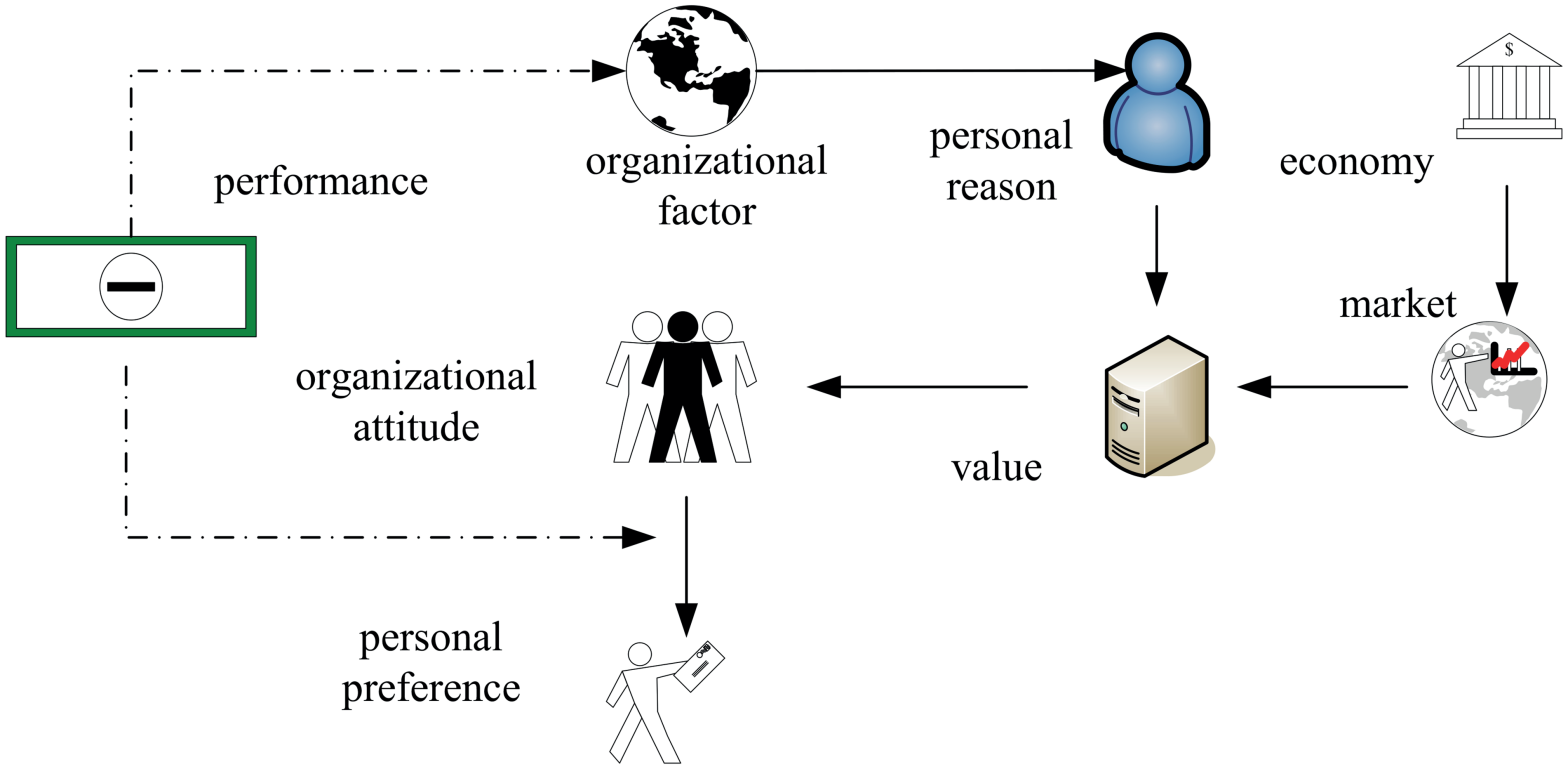
Personal willingness that arises in situations of occupational stress.

With the deepening of psychological research, relevant researchers have discussed the relationship between social and psychological factors in the work environment and health. They believed that work stress can lead to a series of physical and psychological diseases. The factors that lead to occupational stress include environmental conditions; job requirements, including responsibility, load, and rhythm; and labor organization, including management style, interpersonal relationships, and work and rest arrangements. And the personal factors include ability and personal quality. According to relevant research, work stress is divided into three categories, including homework demands, organizational factors, and physical factors of the work environment. The unfavorable physical factors of the working environment increase the overall workload of the staff and reduce the tolerance of stressful factors. A typical case is that group psychogenic diseases often occur in places where the working environment is uncomfortable. Therefore, in order to reduce the occupational stress of employees, it is necessary to provide a more comfortable physical environment (Martinez, 2018; Zhang and Thompson, 2019). Figure 2 shows the personal wishes that employees may have in occupational stress situations.

**Figure 3:**
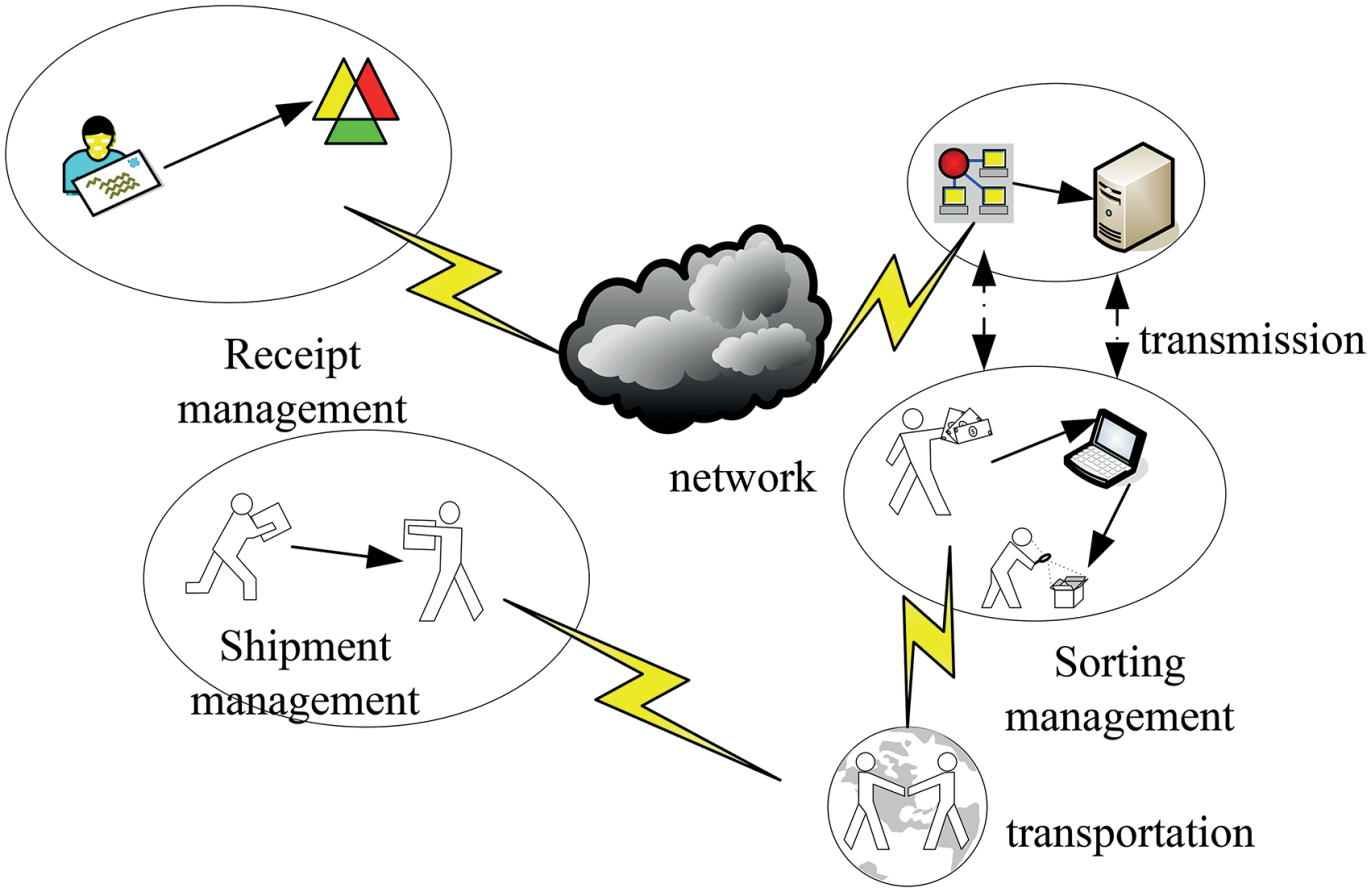
Workflow related to express delivery business.

At the beginning of the last century, the express delivery business emerged in European and American countries. With the continuous development of the economy, the express delivery business continued to expand. In an increasingly competitive environment, it is necessary to provide better services. With the continuous improvement of market rules, express delivery practitioners need to face more and more work pressure. Excessive work pressure will lead to a series of psychological problems for employees. The main features of express delivery include quickness, convenience, security, and networking. And quickness is the most important feature of express delivery services. In order to relieve the work pressure of employees, the express delivery companies need to be optimized. Figure 3 shows the workflow related to the express delivery business.

**Figure 4:**
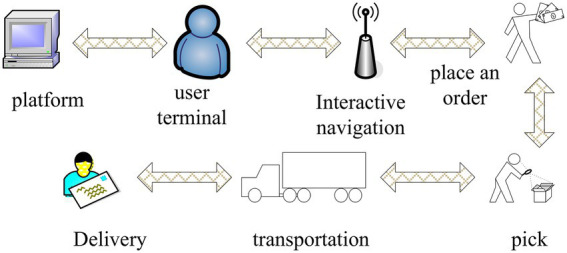
Distribution business structure of logistics express industry.

Human resource allocation can enable enterprises to improve work efficiency, fully realize organizational goals, ensure the normal operation of enterprises, and maximize team strength. In the specific work arrangement, the enterprise needs to determine the number of tasks required according to the prior production technology and task volume, and then arrange the employees according to the relevant needs. In the process of task arrangement, the wishes of employees need to be considered. The reasonable requirements of individual employees can be adjusted without affecting the number of employees required to complete the task. This task arrangement method is also a reasonable allocation of resources, which is a combinatorial optimization problem. Figure 4 shows the distribution business structure of the logistics express industry.


(1) $$W=\min\sum \sum t_{\mathrm{ab}}p_{\mathrm{ab}}$$

Equation 1 is the mathematical model of task assignment. Usually, a matrix was used to represent the solution of the above problem, and the Hungarian algorithm is usually used to solve this kind of problem.


(2) $$\beta =\sum k_{a}$$

In function Equation 2, k represents the object participating in resource allocation, and a represents the relevant number of people.

Before the official start of work, employees need to undergo pre-job training, and the company adopts a fair distribution model for the training and learning places.


(3) $$F=\sum U_{a}$$

In the function Equation 3, U means the seats that can be allocated, and $U_{a}$ means the seats actually allocated by a party.


(4) $$g_{a}=\frac{k_{a}}{\beta }U$$

In Equation 4, U is a positive integer, and $g_{a}$ represents the number of employees assigned to a party.


(5) $$G=\frac{k}{U}$$

The average number of employees per seat in this allocation is G .


(6) $$d=\max\frac{k_{a}}{g_{a}}$$

(7) $$\sum U_{a}=U,R\leq U_{a}\leq k_{a}$$

In order to make the opportunities of employees as fair as possible, each department of the enterprise needs to have a seat. It needs to have $k_{a}$ seat at most, and the final number should be as close to $g_{a}$ as possible.

**Figure 5:**
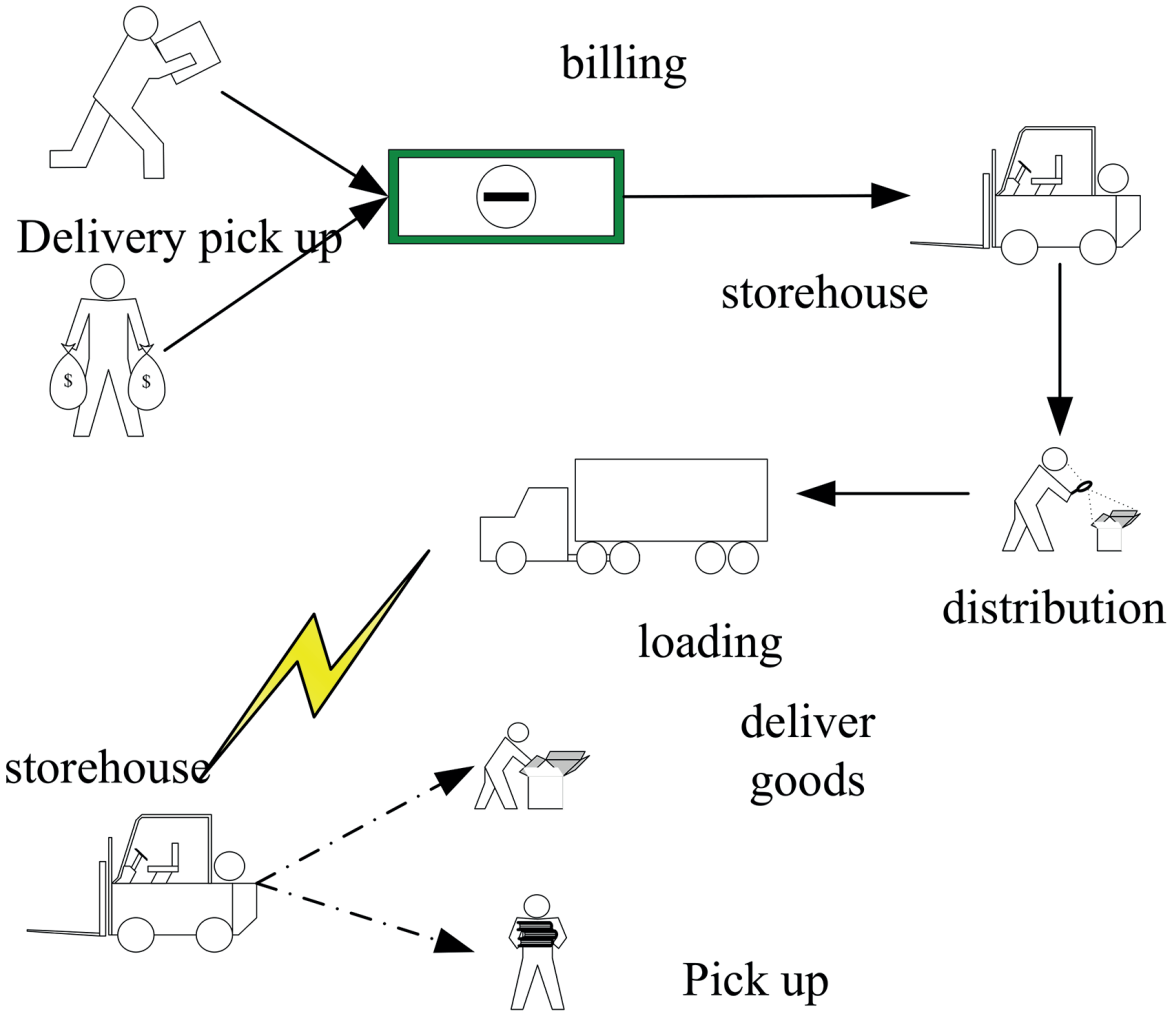
Overall job requirements for the entire express delivery business.

Express distribution outlets are the most basic units of express companies in cities. They are responsible for express transportation and other related work in specific areas. In the final analysis, the express industry belongs to the service field, so its delivery at the end is the most important part of the entire enterprise. The increase in the profit of all express delivery terminals can enhance their own strength and improve their competitiveness in the entire industry. The express delivery industry is affected by seasons, especially during the holidays. E-commerce promotions make people’s consumption fluctuate seasonally. In order to arrange the time reasonably, it is necessary to predict it and find the changing trend of the time series. Figure 5 shows the overall work needs of the entire express delivery business.


(8) $$\bar{r}=\frac{2}{u}\sum \sum p_{\mathrm{ab}},u=zx$$

In the function Equation 8, z represents the number of days of collection, x represents the time period of collection, $p_{\mathrm{ab}}$ represents the sample data, and $\bar{r}$ represents the arithmetic mean of the data of all time periods per day.


(9) $$\bar{r_{b}}=\frac{2}{z}\sum p_{\mathrm{ab}}$$

Equation 9 represents the arithmetic mean of the data in the same time period.


(10) $$l_{b}=\frac{\bar{r_{b}}}{\bar{r}}$$

Equation 10 represents the coefficient for each time period.


(11) $$o_{z+1}=\frac{\sum \partial_{a}\vartheta_{a}}{\sum \partial_{a}}$$

(12) $$\vartheta_{a}=\sum p_{\mathrm{ab}}$$

The function Equations 11 and 12 represent the forecast calculation, and $o_{z+1}$ represents the weighted average of the forecast days.


(13) $${\overset{=}{\vartheta }}_{z+1}=\frac{\vartheta_{z+1}}{x}$$

Among them, $\vartheta_{a}$ represents the total number on the a -th day, and ${\overset{=}{\vartheta }}_{z+1}$ represents the average value of each period of the forecast time.


(14) $$\vartheta_{z+1,b}=l_{b}*{\overset{=}{\vartheta }}_{z+1}$$

Equation 14 represents the predicted value for the b -th time period. Using this forecasting method only needs to give the historical data of a set of predictor variables. It does not need to spend energy to analyze the causal relationship between each variable, but only needs to extend the trend judged by this time series model. Time series analysis is the theory and method of establishing mathematical models through curve fitting and parameter estimation based on time series data obtained from systematic observations.


(15) $$R_{l}=R_{l-2}-\sum v_{l-2}^{\left(s\right)}+\sum R_{l}^{\left(s\right)}$$

Equation 15 represents the vehicles that can be sent out by the express point in the l time period.


(16) $$U_{l}=U_{l-2}-\sum v_{l-2}^{\left(s\right)}+\sum R_{l}^{\left(s\right)}-E_{l}$$

Equation 16 represents the number of available delivery personnel at the express point in the l time period.


(17) $$\varepsilon_{l}^{\left(s\right)}=\frac{\delta_{l}^{\left(s\right)}}{\delta_{l}^{\left(1\right)}+\delta_{l}^{\left(2\right)}+\delta_{l}^{\left(3\right)}}$$

In the function Equation 17, s represents the number of new shipments that need to be sent to customers in each time period, and $\varepsilon_{l}^{\left(s\right)}$ represents the approximate delivery ratio in each region and each time period.

## Experiment on occupational stress of express delivery workers

With the deepening of research on stress, companies in the market place more and more importance on occupational stress. With the rise of psychology, the test of occupational stress began to involve the physical and psychological aspects of employees. Different from physical and chemical factors, occupational stress does not have strict and clear occupational boundaries. Occupational stress is common in the industry, and there are many social factors in the working environment that can lead to occupational stress. The interaction of these factors and individual characteristics can lead to the emergence of a series of psychological problems. With the widespread application of Internet of Things technology and the continuous improvement of market rules, e-commerce is very popular in the industry. It not only provides job opportunities for express delivery workers, but also brings great pressure to them. In order to explore the occupational stress of express delivery workers, a survey on relevant staff was conducted, and the details are as follows:

According to the data in Table 1, 180 express logistics practitioners who participated in the survey were surveyed. According to the specific situation, among the groups participating in the survey, there are 160 boys, accounting for 88.8%, and 20 girls, accounting for 11.2%. It can be seen that most of the staff in the express delivery industry are male. This situation is mainly related to the work content of the express delivery industry. In most cases, the staff needs to carry and collect related items, which has certain requirements on the strength and physical strength of the staff. In terms of age group, there are 2 staff members over 50 years old, up to 1%; 9 staff members between 40 and 50 years old, up to 5%; 77 staff members between 30 and 40 years old, up to 43% %; the staff between 20 and 30 is 88, up to 49%; and the staff under the age of 20 is 4, up to 2%. According to this situation, workers between the ages of 20 and 40 are the absolute main force in the express delivery industry, indicating that the industry is dominated by young labor. The situation is consistent with the market situation, indicating that the selected object is representative of the industry.

**Table 1:** Basic information of courier workers.

Contents	Breakdown	Frequency	Ratio
Differences	Male	160	88.8
	Female	20	11.2
Age group	Older than 50 years old	2	1
	40–50 years old	9	5
	30–40 years old	77	43
	20–30 years old	88	49
	Less than 20 years old	4	2

According to the data in Table 2, the educational background of the courier employees who participated in the experimental investigation has been investigated. According to the specific situation, it is found that 16 employees have a bachelor’s degree or above, accounting for 9%; 90 employees have a college degree, accounting for 50%; 49 employees have a high school education, accounting for 27%; and 25 people with a junior high school education or below, accounting for 14%. According to the situation, the vast majority of the staff’s education is at the college or high school level. It can be seen that the cultural quality of express delivery workers is generally low and the threshold is relatively low, which is also the reason why most staff choose this job. It is precisely because of the low academic qualifications of the staff that they will be nervous about the technical work in the work process and the work involving new knowledge, which will affect their own work effects, and may cause psychological problems in serious cases.

**Table 2:** Survey on the educational attainment of courier workers.

Contents	Breakdown	Frequency	Ratio
Academic qualifications	Bachelor’s degree and above	16	9
	College	90	50
	High School	49	27
	Junior High School and below	25	14

**Figure 6:**
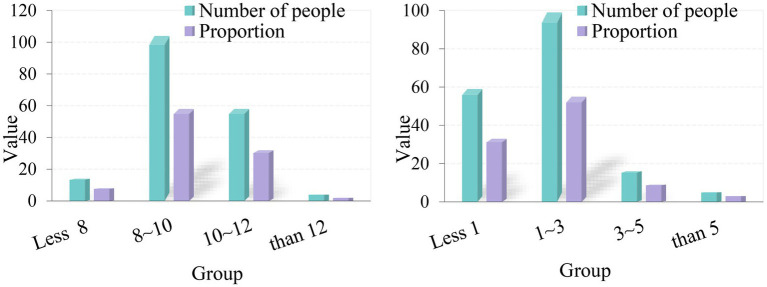
Statistical analysis of working hours and years of express delivery employees.

Occupational stress is mainly related to personal work ability and work content. For the express delivery industry, it is necessary to pay attention to the working hours of the staff. According to the data in Figure 6, the working hours and working years of express delivery employees have been analyzed. First of all, from the perspective of working hours per day, according to the survey, 14 people work less than 8 h a day, up to 8%; 104 people work 8–10 h a day, up to 58%; 58 people work 10–12 h a day, up to 32%; and 4 people work more than 12 h a day, up to 2%. According to the data, the number of workers who work 8–10 h a day is the most, followed by those who work 10 h a day, and the least number of workers who work more than 12 h a day. From this situation, it can be seen that the number of people working for normal hours is very small, and the working hours of most of the staff exceed the standard. It can be seen that the express delivery industry has long working hours and high labor intensity, which may increase the occupational tension of employees. And according to data, the longer the working hours, the higher the occupational tension.

From the perspective of employees’ working years, among the groups participating in the experimental survey, 59 people have worked for less than 1 year, accounting for 33%; 99 people have worked for 1–3 years, accounting for 55%; 16 people have worked for 3–5 years, up to 9%; and 5 people have worked for more than 5 years, up to 3%. Employees with 1–3 years are the most numerous, closely followed by those with less than 1 year. According to this situation, the mobility of employees in the express delivery industry is relatively strong, which is mainly related to the nature of work in the industry.

**Figure 7:**
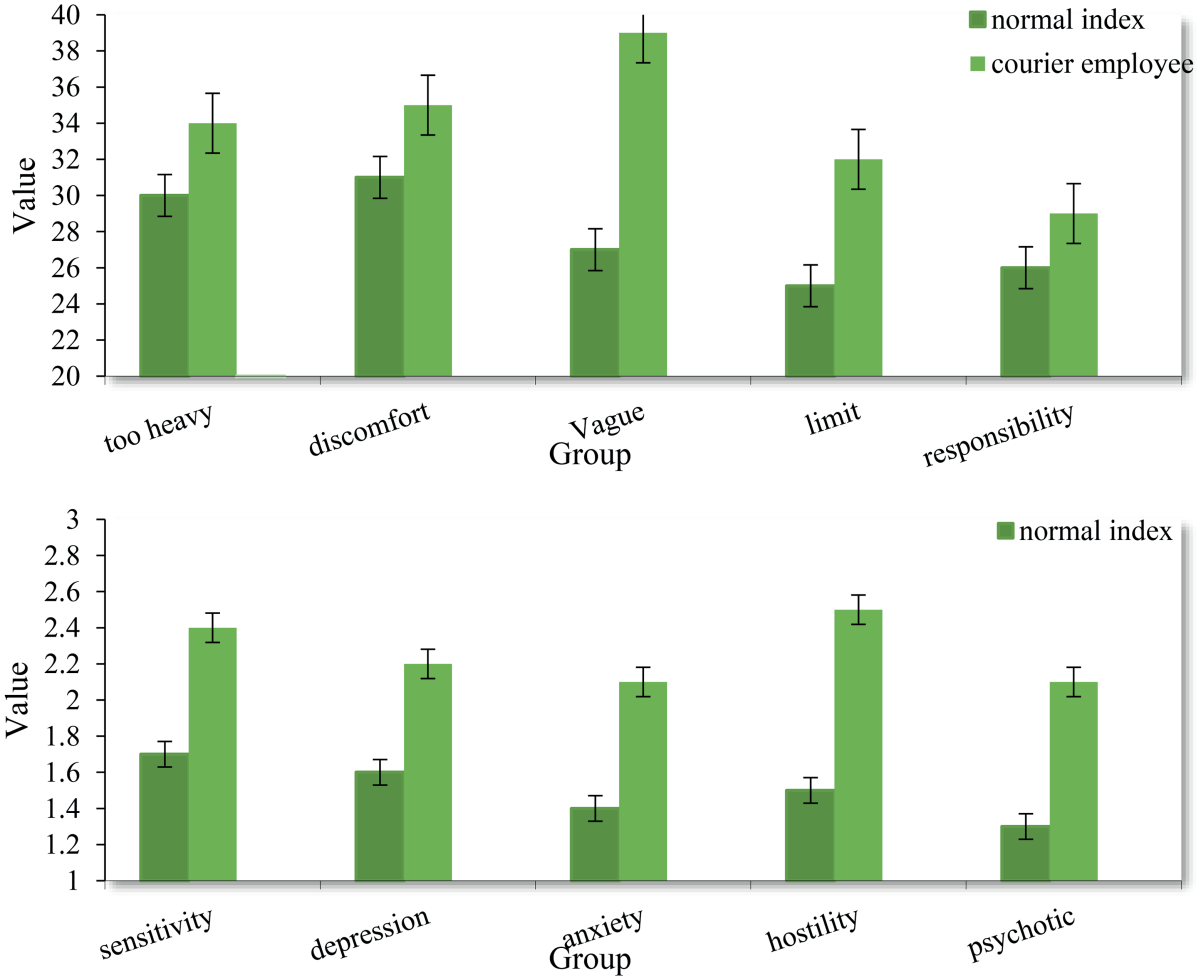
Analysis of occupational stress and mental health level of express delivery workers.

According to the data in Figure 7, the occupational stress and mental health of the staff who participated in the experimental investigation have been analyzed. First of all, from the perspective of occupational stress, when the task is too heavy, the occupational stress index of normal employees is 30, and the occupational stress index of courier employees is 34; when the task is not suitable, the occupational stress index of normal employees is 31, the occupational stress index of normal employees is 35; when the task objectives are too vague, the occupational stress index of normal employees is 27, and the occupational stress index of express delivery employees is 39; when the task boundary is not obvious, the occupational stress index of normal employees is 25, and the occupational stress index of express delivery employees is 32; and when the task requires responsibility, the occupational stress index of normal employees is 26, and the occupational stress index of express delivery employees is 29. According to this situation, compared with normal occupations, the occupational stress index of express delivery workers is higher, especially when the task objectives are too vague. It can be seen that it is necessary to have a clear understanding of the personal capabilities of employees when arranging express delivery services, so as to reduce employees’ professional tension.

From the perspective of employee mental health, according to the data, the interpersonal sensitivity index of employees is 1.7 under normal circumstances, and the index of express delivery employees is 2.4; under normal circumstances, the depression index of employees is 1.6, and the index of express employees is 2.2; under normal circumstances, the anxiety index of employees is 1.4, and the index of courier employees is 2.1; under normal circumstances, the hostility index of employees is 1.5, and the index of courier employees is 2.5; and under normal circumstances, the psychiatric index of employees is 1.3, and the index of express delivery workers is 2.1. According to the data, the mental health index of express delivery employees is higher than the normal index, and the most obvious in terms of interpersonal sensitivity and hostility.

## Mental health correlation of occupational stress among courier workers

E-commerce has brought vitality to the express delivery industry, especially after the Internet promotion. The rise of online shopping has greatly promoted the development of the express delivery industry. While bringing development opportunities to the industry, it also brings challenges to the industry. The workload faced by courier practitioners has increased significantly, especially during holidays and shopping festivals, the staff in the courier industry need to face overloaded work.

According to the data in Table 3, the fatigue of the couriers who participated in the experimental investigation was analyzed and fatigue levels were divided into four levels with A being the least and D being the greatest. According to the specific situation, 6 people have a fatigue level of A, up to 3%; 48 people have a fatigue level of B, up to 27%; 85 people have a fatigue level of C, up to 47%; and 41 people have a fatigue level of D, up to 23%. According to this data, the most fatigued employees are C, followed by B and D. It can be seen that the fatigue level of the vast majority of couriers is relatively high. When the workload of the staff is larger, the emotional fluctuations of the individual are larger, and more psychological problems may occur.

**Table 3:** Investigation on the structure of subjective symptoms of fatigue among couriers.

Contents	Number of people	Proportion
A	6	3
B	48	27
C	85	47
D	41	23

**Figure 8:**
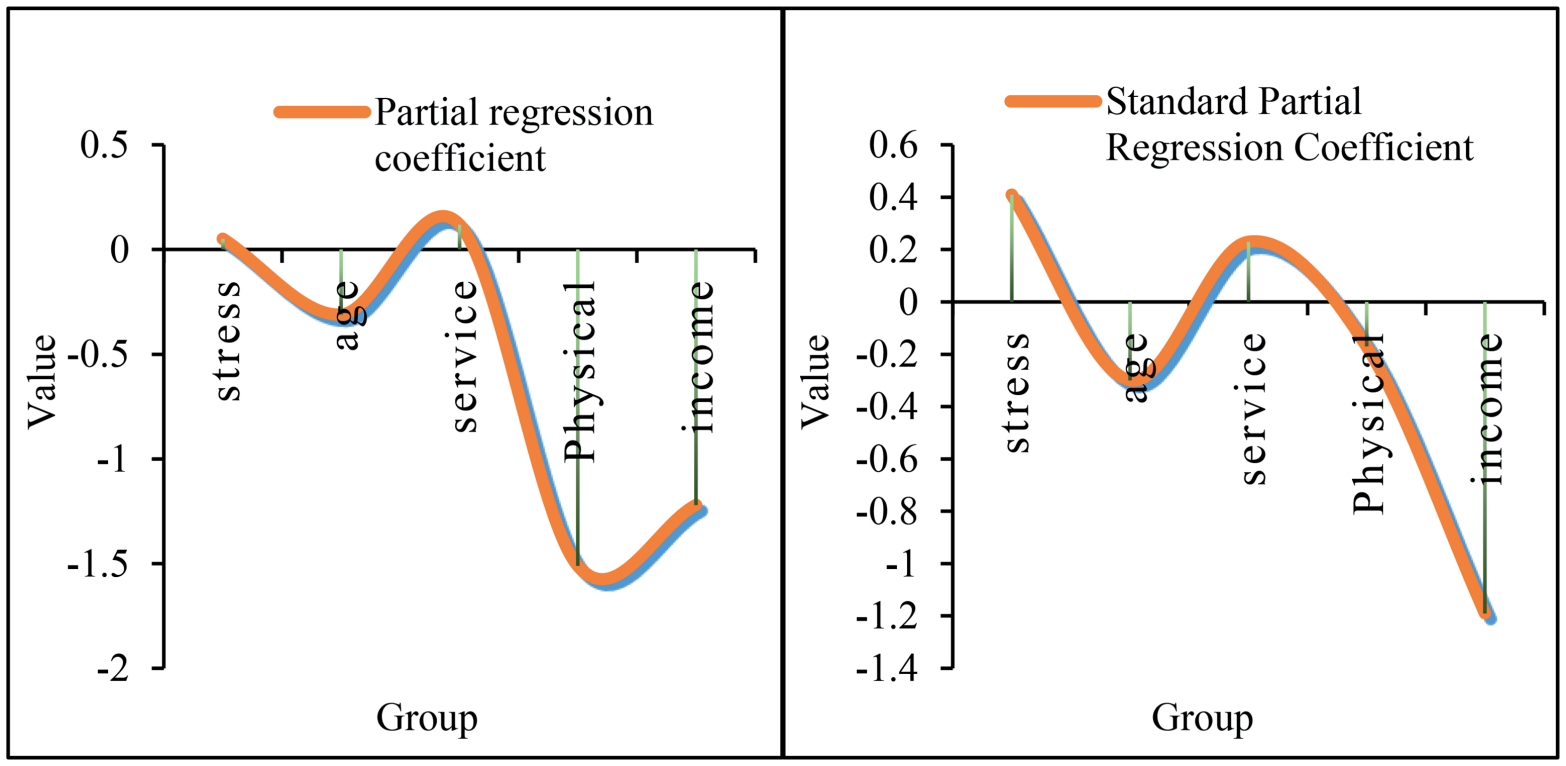
Multivariate stepwise regression analysis affecting interpersonal sensitivity.

According to the above analysis, in the express delivery industry, the interpersonal sensitivity of employees is higher than that in the normal industry, indicating that there is more communication with people in the express delivery industry. According to the data in Figure 8, regression analysis on the multiple factors that affect interpersonal sensitivity has been carried out. First of all, from the partial regression coefficient, the partial regression coefficient indicates the degree of influence of each variable on the sensitivity of randomness to interpersonal relationships. According to the specific data, the coefficient of occupational stress is 0.052, indicating that the degree of occupational stress is proportional to interpersonal sensitivity. The higher the occupational stress, the higher the interpersonal sensitivity index. The coefficient of occupational stress is-0.31, indicating that occupational stress is inversely proportional to age. The larger the value, the lower the interpersonal sensitivity index. The working age index is 0.12, indicating that the degree of working age is proportional to the interpersonal sensitivity and to a certain extent, the greater the working age, the higher the interpersonal sensitivity index. The physical exercise index is-1.51, indicating that physical exercise is inversely proportional to interpersonal sensitivity, indicating that moderate physical exercise can alleviate interpersonal sensitivity. The income index is-1.22, indicating that income and interpersonal sensitivity are inversely proportional. When the individual’s income is higher, the lower the interpersonal sensitivity. According to the standard partial regression coefficient, the occupational stress coefficient is 0.41, and the family income index is-1.19. According to its absolute value, it can be seen that occupational stress has the greatest impact on the interpersonal sensitivity of employees, and family income has the least impact on the interpersonal sensitivity of employees.

**Figure 9:**
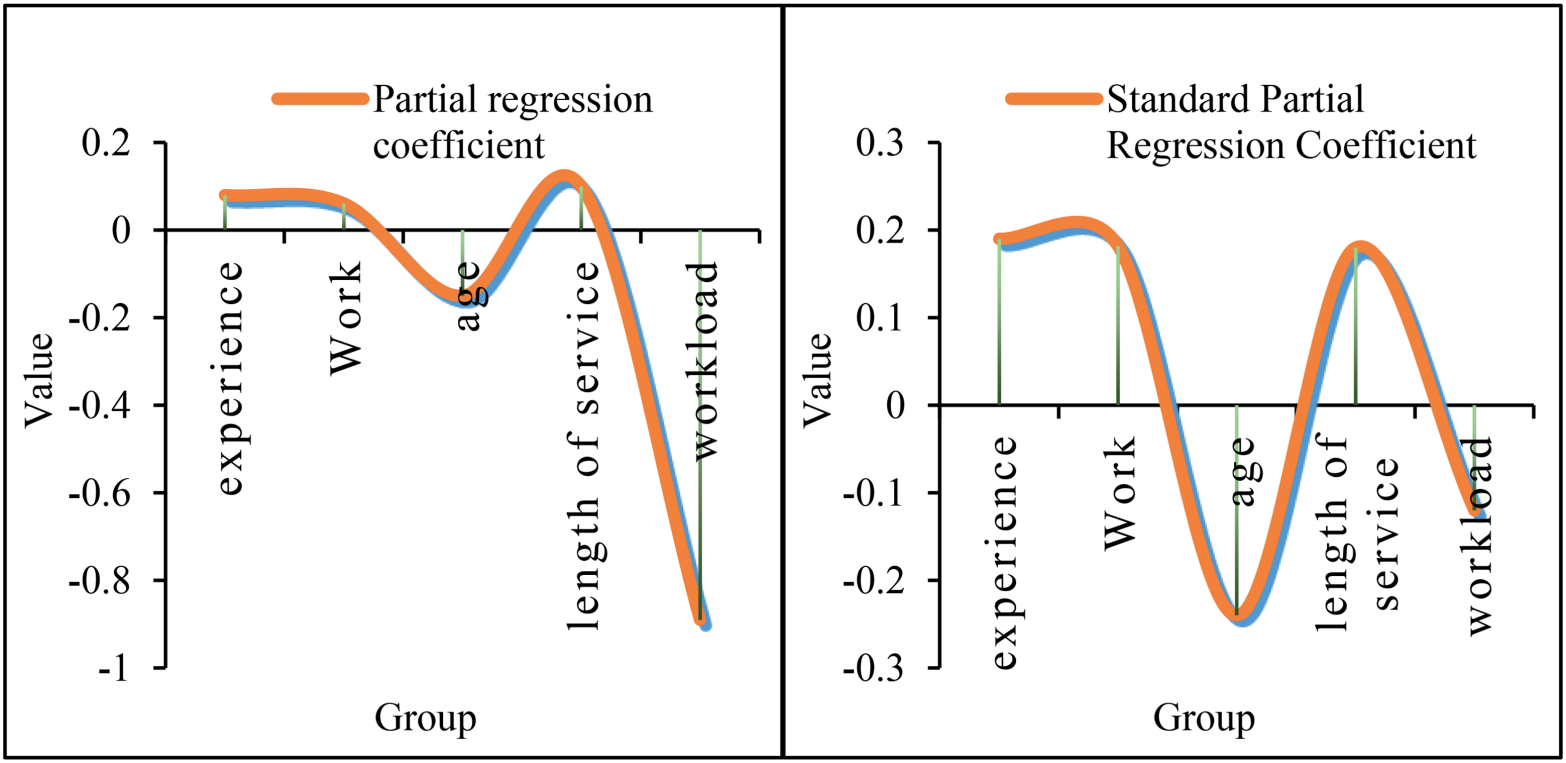
Multi-factor stepwise analysis of the effect of occupational stress on hostile factors.

According to the data in Figure 9, a regression analysis on the multiple factors that affect the hostility has been carried out. First of all, from the perspective of the partial regression coefficient, according to the specific situation, the experience index is 0.08, indicating that the experience is proportional to the hostility. In other words, the richer the experience, the stronger the hostility. The work index is 0.061, indicating that the work is proportional to the hostility, that is, the more skilled the work, the more obvious the hostile consciousness. The age index is-0.15, indicating that the employee’s age is inversely proportional to the hostility, that is, the older the age, the weaker the personal hostility. The length of service index is 0.1, indicating that the length of service is proportional to the hostility. The task volume index is-0.89, indicating that the task volume and hostility are inversely proportional. When the individual’s task volume increases, the focus is more focused, and the less hostile elements are considered. According to the above situation, the relevant workload can be reasonably increased to reduce the hostility of employees. According to the standard partial regression coefficient, the age coefficient is-0.24, and the workload index is-0.12. According to its absolute value, age has the greatest influence on hostile psychology, and the amount of tasks has the least influence on hostile psychology.

## Conclusion

With the promotion of Internet of Things technology, e-commerce has developed rapidly, especially driven by “Internet +,” which has greatly promoted the development of the express delivery industry. While e-commerce brings vitality to the express delivery industry, it also brings a series of challenges to express delivery practitioners. The courier industry has a low entry threshold. In order to obtain higher wages, they usually need to face high-load work and bear huge psychological pressure. Long-term work under high load will not only affect physical health, but also lead to a series of psychological problems. The purpose of this paper is to study the psychological health correlation of express delivery workers’ occupational stress in the information logistics environment. It is expected to explore the relationship between occupational stress and mental health of couriers in the context of informatization, so as to provide employees with a better working environment and reduce personal stress. According to the research, in a certain period, the employee’s working age increases, and the employee’s hostile consciousness is stronger, but the older the employee is, the weaker the employee’s hostile consciousness. Although this paper has drawn some conclusions, there are still shortcomings: the reasons for the occupational stress of couriers of different ages, genders, and working years may be different, but they are not discussed in the paper due to personal knowledge background.

## Data availability statement

The original contributions presented in the study are included in the article/supplementary material, further inquiries can be directed to the corresponding author.

## Author contributions

The author confirms being the sole contributor of this work and has approved it for publication.

## Conflict of interest

The author declares that the research was conducted in the absence of any commercial or financial relationships that could be construed as a potential conflict of interest.

## Publisher’s note

All claims expressed in this article are solely those of the authors and do not necessarily represent those of their affiliated organizations, or those of the publisher, the editors and the reviewers. Any product that may be evaluated in this article, or claim that may be made by its manufacturer, is not guaranteed or endorsed by the publisher.
